# Loneliness, Resilience, Mental Health, and Quality of Life in Old Age: A Structural Equation Model

**DOI:** 10.3389/fpsyg.2017.02003

**Published:** 2017-11-14

**Authors:** Eva Gerino, Luca Rollè, Cristina Sechi, Piera Brustia

**Affiliations:** ^1^Department of Psychology, University of Turin, Turin, Italy; ^2^Department of Pedagogy, Psychology, Philosophy, University of Cagliari, Cagliari, Italy

**Keywords:** old age, loneliness, resilience, mental health, quality of life

## Abstract

**Objectives:** In the scientific literature on aging, a recent core issue has been the role of individuals' internal and external resources, which are considered intrinsically connected, in contributing synergistically to physical and psychological quality of life (QoL). The current study investigates the way in which psychological factors—such as, loneliness, resilience, and mental states, in terms of depression and anxiety symptoms—affect the perceived QoL among elderly individuals.

**Method:** Data from 290 elderly Italian participants were used to study the mediation effects of both mental health and resilience to elucidate the relationship between loneliness and psychophysical QoL.

**Results:** The best model we obtained supports the mediation effect of both resilience and mental health between loneliness and mental and physical QoL. These results highlight that loneliness influences mental and physical QoL via two pathways, with the impact of loneliness mediated by mental health and resilience dimensions.

**Conclusions:** The findings suggest the importance of the support that elderly people receive from social relationships. In terms of clinical interventions, the reduction of loneliness could be an important factor in primary prevention or the recovery process. A way to reduce levels of mental distress could be represented by the increasing of resilience and self-efficacy and reduction of loneliness dissatisfaction. A high degree of resiliency contributes to increasing perceived life quality at the physical and psychological levels, and at the same time, reducing anxiety and depressive symptoms.

## Introduction

In old age, people experience profound changes and face important challenges, including modifications in their roles, retirement, and the death of loved ones (friends and family members). These experiences can increase their levels of stress and lead to a decrease in the resources that individuals feel they have in dealing with their daily lives (Sachs-Ericsson et al., 2014).

In demographic studies, it has been estimated that by 2050, the elderly population in Europe will reach 28%, which emphasizes that the highest proportion of elderly people is currently concentrated on the European continent (Börsch-Supan et al., 2005). This percentage will rise to 34% in Italy by 2025 (United Nations, 2002), while in the last Eurostat report (Eurostat, 2015a), as of 1 January 2014, there were almost 94 million people aged 65 and over in the European Union. The report showed that 16.1% of these people were aged 65–84 years, while 2.4% were 85 years and over. In this scenario, the health condition of the elderly is a core issue; however, although there is a shared recognition of the importance of this aspect at the medical, public, and social levels, unfortunately, it is often a neglected area of scientific study and intervention (National Board of Health and Welfare, Sweden, 2008; Djukanovic et al., 2015; Eurostat, 2015b). As the issues of health, and above all, illness or disability in old age, are a matter of increasing public concern, a perspective on healthy aging is crucial when it comes to identifying, designing, and implementing appropriate strategies to meet the growing needs of the population (Djukanovic et al., 2015).

Considering the projections on the European population aging—especially in Italy—it can be assumed that this issue will become increasingly central in national policies. Lately, as noted by Stephens et al. (2015), the focus of social policies has been changing, shifting from care or symptom reduction to the promotion of well-being according to the biopsychosocial paradigm. The Health Psychology perspective, in opposition to a deficit model, is useful for critically analyzing the effects of strategies to promote healthy aging and reflect on the factors that could improve their efficacy to develop more inclusive models of intervention. Old age is stereotypically considered a period of progressive decline, and consequently, a heavier and heavier healthcare burden for society. This bias in social narratives on aging is traditionally widespread (Jeste et al., 2013; Settersten and Godlewski, 2016), although it has been refuted by experts. For these reasons, and starting from a holistic perspective, there is a growing need for empirical studies that enable the assessment of psychological functioning and overall health in the Third Age (Fry and Debats, 2010; Jeste et al., 2013). Specifically, analyzing the scientific literature, it appears that little is known about the resources that contribute to resilience and well-being in the elderly, as the research has focused more on the weaknesses or dysfunctions in elderly people than on their strengths (Fry and Debats, 2010).

According to the World Health Organization (2002, 2015), quality of life (QoL) can be defined as a subjective perception of the self-positioning in life that combines a person's psychological and PHY—cultural position, value system, expectations, aims and states, independence, and personal beliefs—with the capacity to create relationships. From another viewpoint, the perspective assumed in the theoretical framework of health-related QoL is based on a complex set of relationships that involves biopsychosocial factors related to well-being (Bowling, 2001; Ekwall et al., 2005; Gerino et al., 2015). In line with this, QoL is defined as a multidimensional concept with both objective and subjective factors that refer to general satisfaction with life or its components (Lawton, 1991; Bowling et al., 2002; Arkar et al., 2004). In the context of geriatric psychology and older people's awareness, it is increasingly clear that individuals' internal and external resources are intrinsically connected, and both these aspects contribute synergistically to physical and their psychological well-being (Ryff and Singer, 2000; Fernández-Ballesteros, 2003, 2008; Fry and Debats, 2010).

### Loneliness, mental health, and quality of life

For the analysis of psychological factors that expose the elderly to the risk of malaise, it has been evidenced that depressive symptoms affect the QoL of the elderly population (Beekman et al., 1999; Blane et al., 2008). Concerning adult life, according to Blazer (2003), among the causes of emotional distress, the presence of depressive symptoms is the most frequent, as this condition significantly contributes to decreasing the QoL of the older segment of the population. For example, depressive symptoms have been proven to be associated with functional impairment, chronic diseases, and mortality (Schoevers et al., 2000; Nilsson et al., 2011; Djukanovic et al., 2015). Moreover, researchers have identified close associations between the presence of depressive symptoms and loneliness (Barg et al., 2006; Cacioppo et al., 2006, 2010; Hawkley et al., 2009; Hawkley and Cacioppo, 2010). As indicated by Peplau and Perlman (1982), loneliness can be defined as a set of negative emotional states arising when a subject feels a discrepancy, in an unfavorable direction, between the desired and actual social relationships. Studies have shown that loneliness can be a significant predictor of increases in depressive symptomatology at least 1 year later (Cacioppo et al., 2010).

Although many older people maintain a satisfactory condition of life, risks related to loneliness and psychological distress grow with age (Fry and Debats, 2002). As stated by Fry and Debats (2002), in fact, some elderly people with self-expectancies or internalized beliefs about their aging can experience severe anxiety connected with feelings of loneliness. Clinicians and institutions that are dealing with the elderly have shown a growing concern about its consequences, including profound depressive feelings. Cacioppo et al. (2006) observed that loneliness is associated with strong negative feelings, and other researchers have shown that it impairs self-regulation (Baumeister et al., 2005) or that “[l]onely adults have poor emotion regulation and are less likely to use positive feelings to alleviate their negative mood” (Wong et al., 2016, p. 2487). Loneliness, anxiety, and depressive symptoms may contribute synergistically to a significant decrease in levels of well-being (Liu and Guo, 2007). In addition, Sachs-Ericsson et al. (2014) studied the consequences of rape in an elderly sample, and found a connection between loneliness, depression, anxiety, and psychological functioning. Considering that multiple studies have pointed out that depression and loneliness are strongly associated and that they have detrimental effects on well-being in the Third Age (Tiikkainen and Heikkinen, 2005; Cacioppo et al., 2006; Golden et al., 2009; Theeke, 2010, 2012; Prieto-Flores et al., 2011; LeRoy et al., 2017), it is important to further investigate the prognosis for older persons suffering from depression (Bjørkløf et al., 2013).

### Loneliness, resilience, and QoL

In the general population, people with a low sense of self-efficacy are subject to an increased risk of physical and mental health issues (Marshall, 1991; Krause, 1994; Smith-Osborne and Felderhoff, 2016). In the elderly, loneliness dissatisfaction can significantly contribute to reduce self-evaluations of perceived self-efficacy (i.e., Fry and Debats, 2002). In Bandura's (1977) definition, self-efficacy can be conceptualized as the perception that a person has his/her own ability to enact effective and functional responses to environmental demands. Specifically, this construct refers to people's individual differences in their aptitudes and dispositions when they evaluate themselves as able or unable to cope with situational demands in different contexts and situations (Jerusalem and Schwarzer, 1992). It can be considered a global personality trait, specifically, and permanently connected to the self-perception of mastery (Bandura, 1977, 1982, 1986, 1997, 2000; Luszczynska et al., 2005), and because of its core role in individuals' evaluation of their skills, it is closely linked with the dimension of subjective well-being (Gabriel and Bowling, 2004).

The level of generalized self-esteem is a factor that is interrelated with the dimension of well-being in its physical, emotional, and psychological components (Smith et al., 2000; Bandura, 2004; Fry and Debats, 2010). Longitudinal studies support the view that resilience traits, like self-efficacy, are protective in the later life stage (Smith-Osborne and Felderhoff, 2016) and that these beliefs are linked to stress resistance in the face of minor distress (i.e., anxiety and loneliness; e.g., Fry, 2001; Fry and Debats, 2006, 2010). As pointed out by the American Psychological Association (2004) and Bonanno (2004), resilience is configured as a common response to losses and conditions of severe stress during the lifecycle. Concerning people's ability to deal with adverse conditions in the lifespan, the attention to the construct of resilience progressively increases in relation to QoL in older people (Fry and Keyes, 2010; MacLeod et al., 2016). Gattuso (2003), Braudy Harris (2008), and recently, other authors (Wiles et al., 2012; Stephens et al., 2015), have suggested that the construct of resilience is useful for understanding health in older people. The American Psychological Association (2011) defines resilience as a successful adaptation process in response to threatening, stressful, or traumatic adverse experiences, or the ability to bounce back from difficult life conditions. It is a flourishing state despite adversity (Hildon et al., 2010), where, in the case of the elderly, “adversity” may be considered in terms of an increased frequency of life conditions that entail personal loss, inequalities, disabilities, and the general PHY challenges of aging (Stephens et al., 2015).

Wild et al. (2013) stated that resilience is a key component in successful aging. Several authors have specified that the different generations do not differ in their ability to be resilient (Carstensen et al., 2003; Laditka et al., 2009; Vahia et al., 2012), but MacLeod et al. (2016) stated that resilience may support longevity. Furthermore, according to these authors, high resilience in later life has been associated with positive health outcomes. According to the international scientific literature, it is possible to identify the following outcomes: reduced vulnerability to depressive symptomatology and mortality risks (Sharpley and Yardley, 1999; Carstensen et al., 2003; de Jager et al., 2003; Fredrickson et al., 2003; Inui, 2003; Wallace, 2003; Charney, 2004; DeSalvo et al., 2006; Montross et al., 2006; Reichstadt et al., 2007; Laditka et al., 2009; Lamond et al., 2009); better self-perceptions of aging successfully (Montross et al., 2006); and increased levels of QoL, mental health, and well-being, with improved lifestyle behaviors (Inui, 2003; Montross et al., 2006; Reichstadt et al., 2007; Vahia et al., 2012). According to Connor and Zhang (2006), resilience is a key target of anxiety and depression treatment. Studying the role of the ability to savor positive life experiences in terms of older people's life satisfaction, Smith and Hollinger-Smith (2015) confirmed that people with lower levels of resilience tend to report higher depression. Finally, resilience seems to be a protective factor for depression symptoms in the case of the spousal careers of people with dementia (O'Rourke et al., 2010; O'Dwyer et al., 2013).

To our knowledge, and according to Bowling et al. (2002), the way in which psychological factors—including loneliness, resilience, and individuals' mental states, in terms of depression and anxiety—affect the perceived QoL is still largely unexplored. As described above, authors have studied the psychological variables that can be predictive of QoL, but how variables mediate and influence perceived QoL requires further elucidation.

## Aims

The purpose of the study was to explore a multidimensional model including the relationships among loneliness, resilience, mental health, and mental and physical QoL among elderly individuals. In line with authors who found relationships between loneliness and mental and physical QoL (Tiikkainen and Heikkinen, 2005; Cacioppo et al., 2006; Golden et al., 2009; Theeke, 2010; Prieto-Flores et al., 2011) and those who found relationships among loneliness, resilience, and mental health (Fry and Debats, 2002; Fry and Keyes, 2010; Wild et al., 2013; Sachs-Ericsson et al., 2014), our hypothesis was that higher loneliness levels would be associated with low levels of mental health and resilience, and loneliness, resilience, and mental health would be associated with mental and physical QoL. It was also expected that both resilience and mental health would mediate the negative association between loneliness and mental/physical QoL.

## Materials and methods

### Participants

The sample comprised 290 older adults from Italy (70% females and 30% males), aged 65–90 years (*M*_*Age*_ = 74.7 years, *SD* = 6.9 years); the participants were split into two groups—those in the age range of 65–74 years old (66% females and 34% males) comprised the young old group (*M*_*Age*_ = 69 years, *SD* = 2.9 years), while those older than 74 years (73% females and 27% males) comprised the old group (*M*_*Age*_ = 81 years, *SD* = 4.3 years). Participants volunteered for the study. They were all Italian native speakers, and they were active in the cultural and social activities of the neighborhood. None of the participants were undergoing medical/neurological or psychiatric treatment at the time of assessment. Their education levels were as follows: 58% of the participants had an elementary school education, 31% had a high school diploma, and 11% had completed a university degree. Fifty-one percent of participants were married or in a civil partnership and lived independently with their spouses; 49% of the participants were single, widowed, separated, or divorced and lived alone (40.2%), with their children (6.4%), with brothers or sisters (1.4%), or with other people (e.g., carers; 1%).

### Measures

#### The UCLA loneliness scale (version 3)

The UCLA Loneliness Scale (Version 3) (Russell, 1996; 10 positively worded items [PI] = non-loneliness and 10 negatively worded items [NI] = loneliness) is used to assess participants' level of loneliness, defined by an incongruity between actual and desired social interaction. On this scale, participants are asked to report how often (from 1 = never to 4 = often) they feel the way illustrated for each item. Positive items are reverse coded to generate a global measure in which higher scores denote greater loneliness. For the present study, the authors adapted the scale into Italian using the back-translation technique to guarantee the semantic correspondence of the Italian and English versions. Based on the current participants, the Cronbach's alpha coefficient was 0.87 for global loneliness, 0.83 for the loneliness subscale (NI), and 0.84 for the non-loneliness subscale (PI).

#### The geriatric anxiety inventory—short form

The Geriatric Anxiety Inventory—Short Form (GAI-SF; Byrne and Pachana, 2011) consists of five items, and it is used as a screening tool for individualizing anxiety in older adults. Questions require yes/no answers. It was developed as a briefer version of the full 20-item GAI, and its validity and internal consistency have been demonstrated (Cronbach's alpha 0.81). For the present investigation, the authors adapted the GAI-SF into Italian using the back-translation technique to guarantee the semantic correspondence of the Italian and English versions. Based on the current participants, the Cronbach's alpha coefficient was 0.78.

#### The geriatric depression scale

The Geriatric Depression Scale—Short Form (GDS-SF; Hoyl et al., 1999; Italian version, Rinaldi et al., 2003) consists of five items, and it is used as a screening tool for individualizing depression in older adults. It comprises items about how the person has felt over the past week. The questions require yes/no answers. It was developed to be a version of the 15-item GDS, and its overall performance has been demonstrated to be comparable to that of the 15-item scale. Moreover, the 5-item GDS is a better screening tool than the 15-item version is (Hoyl et al., 1999). Based on the current participants, the internal consistency coefficient was 0.70.

#### The generalized self-efficacy scale

The Generalized Self-efficacy (GSE) Scale (Jerusalem and Schwarzer, 1986; Italian version, Sibilia et al., 1995) consists of 10 items, and it is designed to measure a sense of perceived self-efficacy with the objective of predicting coping with everyday adversity, as well as adjustment after experiencing different types of stressful life events. The participant responds to the instrument using a 4-point Likert scale (from 1 = not at all true to 4 = exactly true). A high score signifies a high perception of self-efficacy. The GSE has been administered widely and has been found to have satisfactory internal consistency reliability. Based on the current participants, the internal consistency coefficient was 0.87.

#### The resilience scale

The Resilience Scale (RS; Wagnild and Young, 1993; Italian version Peveri, 2010) consists of 10 items rated on a 7-point Likert scale (from 1 = disagree to 7 = agree), and it is a measure of the ability to bear stressful life events and make meaning from challenges. The concurrent validity and internal consistency reliability of the RS scale have been shown to be adequate (Wagnild, 2009). Based on the current participants, the internal consistency coefficient was 0.91.

#### The world health organization quality of life questionnaire

The World Health Organization QoL (WHOQOL-BREF; World Health Organization, 1993; Italian version, De Girolamo et al., 2000) Questionnaire evaluates QoL in four areas, as follows: psychological health (PSY), physical health (PHY), environment (E), and social relationships (SR). It includes 24 self-report items, and the participant responds to the instrument via a 5-point Likert scale (from 1 = not at all to 5 = completely). It is a shorter version of the original tool, and it may be better adapted for use in big clinical trials or studies. Higher scores show a higher perceived QoL. The WHOQOL-BREF is appropriate for use with older adults (Lucas-Carrasco, 2012). For this study, the subscales assessing QoL across the physical and PSY domains were used. Based on the current participants, the internal consistency coefficient was 0.85 for the PHY subscale and 0.78 for the PSY subscale.

### Procedure

All participants were contacted individually at their place of living and signed the written informed consent. Participants who agreed to participate, understood the instructions, and met the selection criteria autonomously completed a questionnaire on demographic data, the UCLA, GAI-SF, GDS-SF, GSE, RS, and WHOQOL–BREF self-reports. The questionnaires were offered in a counterbalanced order on two forms, and no order effect was found. The confidentiality of participants' answers was guaranteed. The time needed to fill in the questionnaires was approximately 60 min.

### Data analyses

Descriptive statistics were computed on the evaluated psychological variables, reporting means, standard deviations, kurtosis, and skewness. The association between continuous variables was tested by means of Pearson correlations. A two-step process was adopted to test the hypothesized model, as follows: (1) a confirmatory factor analysis was implemented to create a measurement model with an adequate fit to the data; and (2) the structural equation model (SEM) established after this phase was verified in the second step (Anderson and Gerbing, 1988). The hypothesized model comprised four latent factors (loneliness, mental health, resilience, and mental and physical QoL) and eight observed variables. Specifically, it comprised one hypothetical latent independent factor, two latent mediator factors (mental health and resilience), and one latent dependent factor (mental and physical QoL). The loneliness latent factor was measured using the two subscales of UCLA (PI and NI). The mental health latent factor was measured using two sources, the GAI-SF and GDS-SF, while the resilience latent variable was measured by the GSE and RS. As mentioned above, the concept of resilience is a multifaceted construct, and together, these scales may provide a more complete assessment of resilience than each measure alone would. Finally, the mental and physical QoL latent factor was measured using two subscales of the WHOQOL–BREF (PHY and PSY).

For evaluating the model fit, a set of fit indices were used based on recommended criteria, including the following: a comparative fit index (CFI) and Tucker–Lewis index (TLI) ≥0.90, which showed an acceptable fit of the model (Bentler, 1990; Schumacker and Lomax, 1996; Kline, 2005; Brown, 2006); the root mean square error of approximation (RMSEA), where values ≤0.05 can be regarded as an appropriate fit and values between 0.05 and 0.08 as an acceptable fit (Browne and Cudeck, 1993; Hu and Bentler, 1999; Brown, 2006); and the standardized root mean square residual (SRMR) of <0.1 (Bentler, 1990).

To compare the models, the Akaike information criterion (AIC; Akaike, 1987) of smaller values representing a better fit of the hypothesized model (Byrne, 2001) and expected cross-validation index (Browne and Cudeck, 1993) of the smallest values exhibiting the greatest potential for replication (Byrne, 2001) were also considered to establish the best model. Finally, to establish whether the hypothesized model performed equivalently across age, multi-group analyses were run.

## Results

### Descriptive analysis

Descriptive statistics of eight observed variables were tested to check for the normality of distribution. For each of the observed variables, the kurtosis and skewness values were between 1 and −1; therefore, this sample can be defined as having a normal distribution. The descriptive statistics of the eight observed variables are shown in Table 1. Correlations were computed to study the relationships of all eight measured continuous variables. The coefficients of correlation are shown in Table 2. The results revealed that loneliness is significantly and positively correlated with anxiety and depression and negatively associated with resilience, self-efficacy, and psychological and PHY.

**Table 1:** Means, standard deviations, skews, and kurtosis for eight observed variables.

**Variable**	**M**	***SD***	**Skewness**	**Kurtosis**
PI	28.4	4.4	−0.7	1
NI	24.6	6	0.3	0.1
GAI-SF	1.7	1.7	0.6	−0.9
GDS-SF	1.2	1.2	1	0.8
RS	54.9	9	−0.9	1
GSE	27.7	4.1	−0.4	0.5
PSY	80.7	13.1	−0.5	0.8
PHY	101.7	18	−0.6	0.6

*PI, positively worded items of UCLA Loneliness Scale-3; NI, negatively worded items negatively worded items; GAI-SF, Geriatric Anxiety Inventory—short form; GDS-SF, Geriatric Depression Scale; RS, Resilience Scale; GSE, Generalized Self-Efficacy Scale; PSY, psychological health subscale of WHOQOL-BREF-World Health Organization Quality of Life Questionnaire; PHY, physical health subscale of WHOQOL-BREF- World Health Organization Quality of Life Questionnaire*.

**Table 2:** Pearson correlations for the eight observed variables.

	**1**	**2**	**3**	**4**	**5**	**6**	**7**	**8**
1.PI	1							
2.NI	0.403^**^	1						
3.GAI-SF	0.288^**^	0.268^**^	1					
4.GDS-SF	0.368^**^	0.330^**^	0.447^**^	1				
5.RS	−0.394^**^	−0.422^**^	−0.388^**^	−0.498^**^	1			
6.GSE	−0.160^**^	−0.238^**^	−0.335^**^	−0.314^**^	0.629^**^	1		
7.PSY	−0.431^**^	−0.410^**^	−0.531^**^	−0.559^**^	0.676^**^	0.529^**^	1	
8.PHY	−0.289^**^	−0.302^**^	−0.387^**^	−0.439^**^	0.509^**^	0.410^**^	0.638^**^	1

** *p<0.01*.

### Mediation model

#### First phase: measurement model

**Figure 1:**
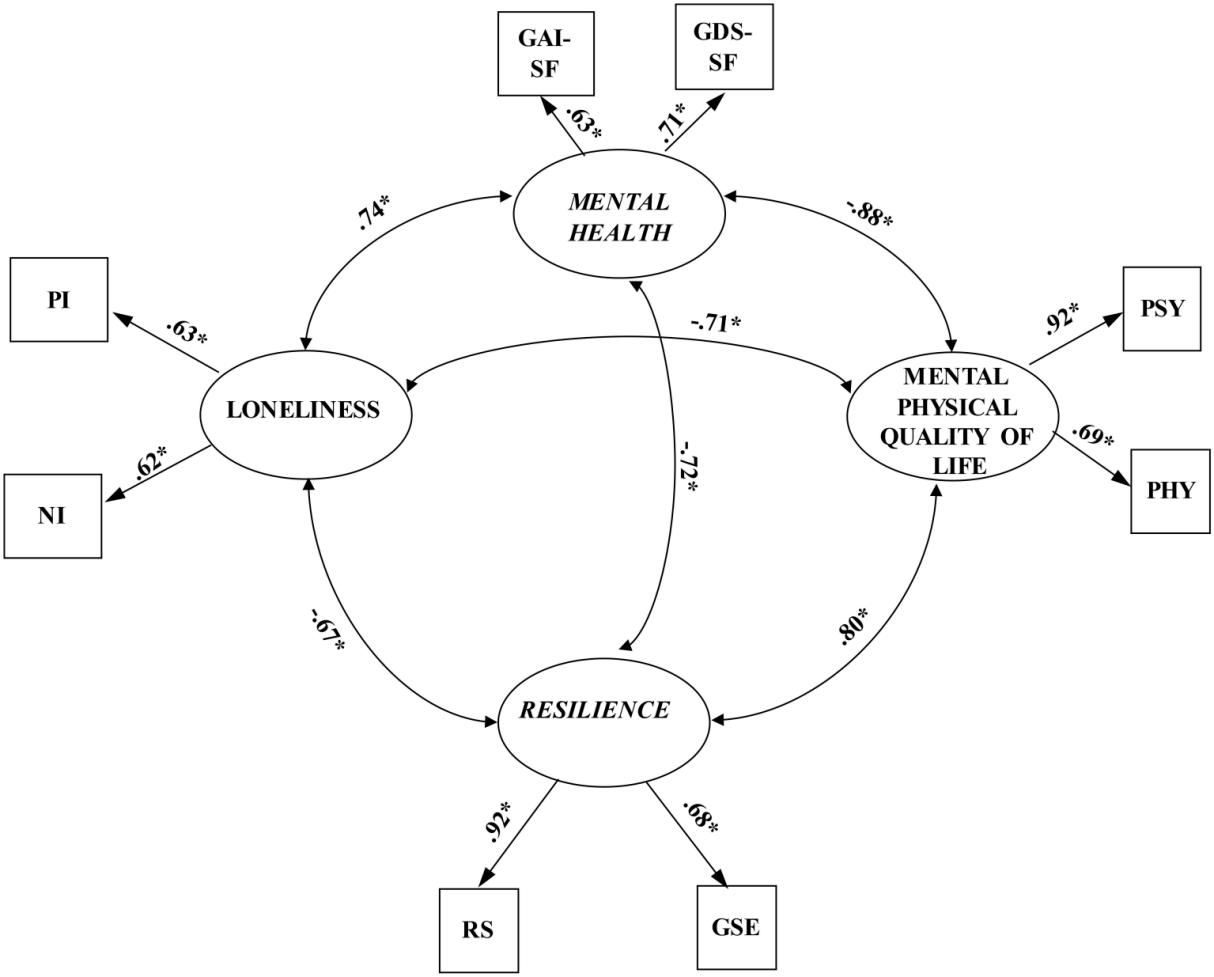
The measurement model (*N* = 290). Factor loadings are standardized. PI, positively worded items of UCLA *Loneliness Scale-3*; NI, negatively worded items negatively worded items; GAI-SF, Geriatric *Anxiety Inventory—short form*; GDS-SF, *Geriatric Depression Scale;* RS, *Resilience Scale;* GSE, *Generalized Self-Efficacy Scale;* PSY, psychological health subscale of *WHOQOL-BREF- World Health Organization Quality of Life Questionnaire;* PHY, *physical* health subscale of *WHOQOL-BREF- World Health Organization Quality of Life Questionnaire*. ^*^p < 0.001.

The confirmatory factor analysis measured four latent factors (loneliness, mental health, resilience, and mental and physical QoL) and eight observed variables (Figure 1). All latent factors were found to associate with one another. The model was assessed using the method of maximum likelihood. A test of the measurement model showed a very acceptable fit to the data, χ^2^ = 27.80, *df* = 14 *p* = 0.05, CFI = 0.99, TLI = 0.98, RMSEA = 0.05 [90% confidence interval (CI): 0.01–0.08], SRMR = 0.03. In addition, all the factor loadings were significant, *p* < 0.001, which supports the convergent validity of the indicators (Anderson and Gerbing, 1988). These results indicate that all the latent factors were well exemplified by their observed variables. In addition, the four latent factors were significantly connected, *p* < 0.001. Thus, this model was used to examine the hypothetical structural model.

#### Second phase: the structural equation model

The SEM was verified using the method of maximum likelihood. To obtain the best model, five alternative models were calculated (Table 3). First, a partially mediated model (Model A) with two mediators and a direct path from loneliness to mental and physical QoL showed an appropriate fit, χ^2^ = 29.85, *df* = 15 *p* = 0.01, CFI = 0.98, TLI = 0.97, RMSEA = 0.06 (90% CI: 0.03–0.08), SRMR = 0.04. However, it is important to note that there was no significant direct effect of loneliness mental and physical QoL in this model, *b* = 0.11 *p* > 0.05. Thus, a fully mediated model (Model B) was verified with this path constrained to zero, which showed a good fit to the data, χ^2^ = 29.97, *df* = 16 *p* = 0.02, CFI = 0.98, TLI = 0.97, RMSEA = 0.05 (90% CI:0.02–0.08), SRMR = 0.03.

**Table 3:** Fit indices among the competing models.

	**Model A**	**Model B**	**Model C**	**Model D**	Model E^*^
χ^2^	29.85	29.97	23.84	26.66	23.84
*df*	15	16	15	16	15
CFI	0.98	0.98	0.99	0.99	0.99
TLI	0.97	0.97	0.98	0.98	0.98
RMSEA	0.06	0.05	0.05	0.05	0.05
CI for RMSEA	0.03–0.08	0.02–0.08	0.00–0.08	0.01–0.08	0.00–0.08
SRMR	0.04	03	03	03	03
AIC	71.85	71.85	65.84	66.66	65.84
ECVI	0.25	0.25	0.23	0.23	0.23
CI for ECVI	0.21–0.32	0.21–0.32	0.20–0.29	0.20–0.29	0.20–0.29

*N = 290*.

* *Represents the best model*.

*CFI comparative fit index, TLI Tucker Lewis Index, RMSEA root-mean-square error of approximation, SRMR standardized root-mean square residual, AIC Akaike information criterion, ECVI expected cross validation index, CI confidence interval*.

Comparing the chi-square differences, no significant difference between Model A and Model B, Δχ^2^ = 0.12, *df* = 1, *p* > 0.05, showing that the Model B exhibited a better fit for the data. Next, a path from mental health to resilience was added to the fully mediated model (Model C), and the results showed an extremely satisfactory fit to the data, χ^2^ = 23.84, *df* = 15 *p* = 0.07, CFI = 0.99, TLI = 0.98, RMSEA = 0.05 (90% CI: 0.0–0.08), SRMR = 0.03. Comparing Model B to Model C, Δχ^2^ = 6.13*, df* = 1, *p* = 0.01, it was shown that the added path contributed significantly to the model. The path coefficient from mental health to resilience was significant, *b* = −0.51, *p* < 0.01; however, the path from loneliness to resilience became non-significant, *b* = −0.28, *p* > 0.05. Thus, this path was eliminated, and the model was retested (Model D). The results also showed an extremely appropriate fit to the data, χ^2^ = 26.66, *df* = 16 *p* = 0.05, CFI = 0.99, TLI = 0.98, RMSEA = 0.05 (90% CI: 0.01–0.08), SRMR = 0.03. However, the chi-square difference between Model C and Model D was not significant, Δχ^2^ = 2.82*, df* = 1, *p* > 0.05, suggesting that Model D was better.

Finally, Model E was verified by adding a path from resilience to mental health to Model B, which showed a good fit to the data, χ^2^ = 23.84, *df* = 15 *p* = 0.07, CFI = 0.99, TLI = 0.98, RMSEA = 0.05 (90% CI: 0.0–0.08), SRMR = 0.03. The standardized path coefficients from resilience to mental health, *b* = −0.40, *p* < 0.01, from loneliness to mental health, *b* = 0.49, *p* < 0.001, and from loneliness to resilience, *b* = −0.66, *p* < 0.001, were significant.

**Figure 2:**
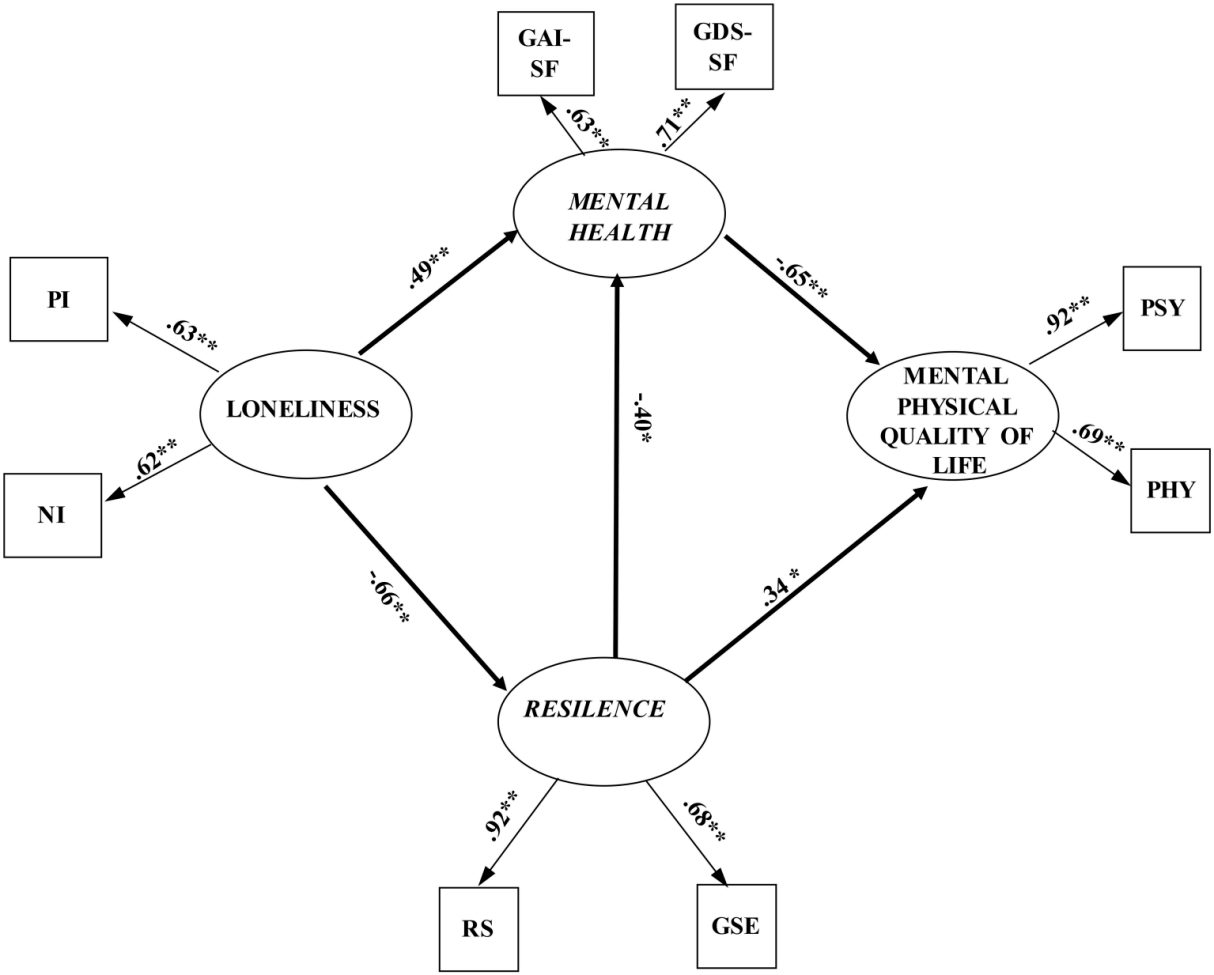
The finalized structural model (*N* = 290). PI, positively worded items of UCLA *Loneliness Scale-3*; NI, negatively worded items negatively worded items; GAI-SF, Geriatric *Anxiety Inventory—short form*; GDS-SF, *Geriatric Depression Scale;* RS, *Resilience Scale;* GSE, *Generalized Self-Efficacy Scale;* PSY, psychological health subscale of *WHOQOL-BREF–World Health Organization Quality of Life Questionnaire;* PHY, *physical* health subscale of *WHOQOL-BREF-World Health Organization Quality of Life Questionnaire*. ^*^p < 0.01 ^**^p < 0.001.

The chi-square difference between Model D and Model E was not significant, Δχ^2^ = 2.82*, df* = 1, *p* > 0.05, implying that Model E was a better model. Furthermore, the slightly smaller AIC value (see Table 3) implied that Model E was better than Model D. Therefore, Model E was designated as the best model (Figure 2). In Model E, mental health and resilience fully mediated the link between loneliness and mental and physical QoL. The partial mediation effect of mental health in the link between resilience and mental and physical QoL was significant. Moreover, resilience partially mediated the link between loneliness and mental health (z = −2.98, *p* < 0.001). Especially, the path of loneliness → resilience → mental health → mental, and physical QoL was significant. This path indicated that elderly people with high loneliness levels are not able to face the adversity, trauma, and stress, which may lower their mental health, and in turn, lead to low mental and physical QoL.

Finally, the multigroup analysis was tested to investigate whether the path coefficients were moderated by age. The age differences (young old group and old group) were tested by comparing the first model, which allows the structural paths to vary across ages, with the second model, which constrains the structural paths across ages to be equal. All the factor loadings, structure co-variances, and error variances were constrained to be equal.

The non-significant chi-square differences between the two models, Δχ^2^ = 8.43, *df* = 6, *p* > 0.05, as well as the slightly smaller AIC value, suggested that the structural paths of the final model did not differ by age, offering initial support for its robustness.

## Discussion

The current study was planned to test the mediation effects of both resilience and mental health for the relationship between loneliness and mental and physical QoL with a sample of elderly Italian people, given the lack of national and international literature concerning a multidimensional model of QoL, loneliness, resilience, and mental health. The best model from the current study supports the mediation effect of both resilience and mental health between loneliness and mental and physical QoL.

These results strongly suggest that loneliness influences mental and physical QoL via two pathways, with the impact of loneliness mediated by mental health and the impact of loneliness mediated by resilience. In other words, elderly people with high levels of loneliness are at an increased risk of experiencing low levels of mental health and low capacity to withstand stressors, resulting in low mental and physical QoL. The QoL seems to be the outcome of different psychological processes interrelated in a complex way, and not a direct effect of the perceived loneliness level.

Another relevant finding of the study regards the path of loneliness → resilience → mental health → mental and physical QoL, which was shown to be significant. This path could underline that elderly people with high loneliness levels are not able to face adversity, trauma, or stress; persons in this condition may evidence a lower resilience level, which may threaten their mental health. In turn, this condition could lead to a lower mental and physical QoL. That is, mental health is a mediator between resilience and mental and physical QoL, while resilience partially mediates the relationship between loneliness and mental health.

In line with the literature (Fry and Debats, 2010), the results of our study seem to indicate that people with greater levels of self-efficacy and resilience can mobilize emotional and psychological resources to face the stressful elements of their lives, and therefore, to express and feel more QoL satisfaction. According to the socio-cognitive model of health proposed by Bandura (1977, 1986, 1988), the concept of self-efficacy is included in a perspective that considers people as having an active role in producing and giving meaning to their experiences. These agency beliefs would affect the way in which the elderly face typical limitations and loss at their stage of life. In fact, according Lawton et al. (1999), maintaining a sense of agency can help a person to preserve a positive attitude toward life, moderating the emotional effect of loneliness and distress and supporting a greater life satisfaction.

In terms of clinical interventions, the model highlights how important the support that elderly people receive from social relationships could be. The reduction of loneliness dissatisfaction may be an important factor in primary prevention or the recovery process. Elderly people's active participation in social activities in their communities could be increased via specific initiatives aimed at the elderly population. The opportunity to reduce the level of mental distress has been evidenced in the model with increasing resilience and self-efficacy and reduced loneliness dissatisfaction. This reduction will strengthen the capacity to face adversity, different losses, and stressful situations (resilience). In addition, as evidenced by the literature, a high degree of resiliency contributes to increased perceived life quality at the physical and psychological levels, and at the same time, reduces anxiety and depressive symptoms. The fact that loneliness could be reduced (Findlay, 2003; Cattan et al., 2005; Fokkema and Van Tilburg, 2007; Dickens et al., 2011; Forsman et al., 2011; Masi et al., 2011; Hagan et al., 2014), self-efficacy beliefs modified (Bandura, 1993), and resilience strengthened (Hartling, 2008) makes these factors primary for early intervention in support of QoL among the elderly (Fry and Debats, 2002). Ultimately, the results could also have economic implications in term of reducing healthcare costs (Bramley et al., 2002) and resulting in fewer contacts between elderly people and general practitioners and hospitals.

### Limits and future perspectives

Critically analyzing the outcomes of the present study, it could be interesting to consider the results in the context of the study's limitations. First, self-report tools were used, and they are not exempt from limitations, such as, inaccurate reporting and social desirability bias. Second, the participation in the study was voluntary; consequently, the sample composition may not represent the characteristics of the general Italian population. Third, the did not considered the variables of being in a couple (Ha, 2016), having siblings (Cicirelli, 2013), or being in a twin relationship (Brustia et al., 2013; Prino et al., 2016).

Future studies should examine and consider the relationship between mental health—in terms of anxiety, depression, resilience, and QoL—from a longitudinal perspective. For example, research could compare people's conditions at different stages in the Third Age or monitor longitudinal changes in the relationships between these factors in the lifecycle. Moreover, they could analyze people resilience and self-efficacy before and after completing a specific intervention program. It would also be interesting to further analyze the absence of the relationship between loneliness and QoL and to carry out the following: (1) pre–post evaluation of a specific training program on increasing resilience and reducing loneliness in a group of elderly people to see if their QoL increases (Lloyd et al., 2017); (2) consider the variable of being in a couple or whether the person a caregiver everyday life; and (3) consider older people who have experienced specific Third Age losses and study the evolution of the mediation model presented here.

## Ethics statement

This research project has been approved by Scientific Commission of “Fondazione Giovanni Goria” and everything has been done in accordance with the ethical standard of Associazione Italiana di Psicologia and with the 1964 Helsinki Declaration and its later amendments or comparable ethical standards. Informed sheet on the questionnaire was given and discussed with all individual participants included in the study and no identifying details (name, surname, dates of birth, identity numbers, and other information) of the participants that were studied has been gathered and collected. After the discussion on the information sheet and to have answered to the their questions about the issues on the administration/questionnaire, each participant gave oral consent before filling. Researchers don't have any opportunity to identify any specific participant.

## Author contributions

EG prepared the study design, organized the sample recruitment, collected data, and contributed to the writing of the manuscript's introduction, discussion, and references sections. LR contributed to the study design and writing of the manuscript's introduction, discussion, and references sections. CS prepared the data set, performed statistical analysis, prepared the tables, and contributed to the writing of the methods and results sections. PB prepared the study design and supervised the research team.

### Conflict of interest statement

The authors declare that the research was conducted in the absence of any commercial or financial relationships that could be construed as a potential conflict of interest.

## References

[B1] Akaike H. (1987). Factor analysis and AIC. Psychometrika 52, 317–332. 10.1007/BF02294359

[B2] American Psychological Association (APA) (2004). Fostering Resilience in Response to Terrorism: For Psychologists Working with Older Adults. Avaliable online at: http://www.apa.org/pi/aging/resources/older-adults.pdf

[B3] American Psychological Association (APA) (2011). The Road to Resilience. Avaliable online at: http://www.apa.org/helpcenter/road-resilience.aspx

[B4] Anderson J. C. Gerbing D. W. (1988). Structural equation modeling in practice: a review and recommended two-step approach. Psychol. Bull. 103, 411–423. 10.1037/0033-2909.103.3.411

[B5] Arkar H. Sari Ö. Fidaner H. (2004). Relationships between quality of life, perceived social support, social network, and loneliness in a turkish sample. Yeni Symp. 42, 20–27.

[B6] Bandura A. (1977). Self-efficacy: toward a unifying theory of behavioral change. Psychol. Rev. 84, 191–215. 10.1037/0033-295X.84.2.191 847061

[B7] Bandura A. (1982). Self-efficacy mechanism in human agency. Amer. Psychol. 37, 122–147. 10.1037/0003-066X.37.2.122

[B8] Bandura A. (1986). Social Foundations of Thought and Action: A Social Cognitive Theory. Englewood Cliffs, NJ: Prentice Hall.

[B9] Bandura A. (1988). Self-efficacy conception of anxiety. Anxiet. Res. 1, 77–98. 10.1080/10615808808248222

[B10] Bandura A. (1993). Perceived self-efficacy in cognitive development and functioning. Educ. Psychol. 28, 117–148. 10.1207/s15326985ep2802_3

[B11] Bandura A. (1997). Self-efficacy: The Exercise of Control. New York, NY: Freeman.

[B12] Bandura A. (2000). Self-efficacy: the foundation of agency, in Control of Human Behaviour, Mental Processes and Consciousness, eds Perrig W. J. Grob A. (Mahwak, NJ: Erlbaum), 17–33.

[B13] Bandura A. (2004). Health promotion by social cognitive means. Health Educ. Behav. 31, 143–164. 10.1177/1090198104263660 15090118

[B14] Barg F. K. Huss-Ashmore R. Wittink M. N. Murray G. F. Bogner H. R. Gallo J. J. (2006). A mixed-methods approach to understanding loneliness and depression in older adults. J. Gerontol. B Psychol. Sci. Soc. Sci. 61, S329–S339. 10.1093/geronb/61.6.S329 17114313 PMC2782769

[B15] Baumeister R. F. DeWall C. N. Ciarocco N. J. Twenge J. M. (2005). Social exclusion impairs self-regulation. J. Pers. Soc. Psychol. 88, 589–604. 10.1037/0022-3514.88.4.589 15796662

[B16] Beekman A. F. Copeland J. M. Prince M. J. (1999). Review of community prevalence of depression in later life. Br. J. Psychiatry 174, 307–311. 10.1192/bjp.174.4.307 10533549

[B17] Bentler P. M. (1990). Comparative fit indexes in structural models. Psychol. Bull. 107, 238–246. 10.1037/0033-2909.107.2.238 2320703

[B18] Bjørkløf G. H. Engedal K. Selbaek G. Kouwenhoven S. E. Helvik A.-S. (2013). Coping and depression in old age: a literature review. Dement. Geriatr. Cogn. Disord. 35, 121–154. 10.1159/000346633 23392253

[B19] Blane D. Netuveli G. Montgomery S. M. (2008). Quality of life, health and physiological status and change at older ages. Soc. Sci. Med. 66, 1579–1587. 10.1016/j.socscimed.2007.12.021 18241966

[B20] Blazer D. G. (2003). Depression in late life: review and commentary. J. Gerontol. A Biol. Sci. Med. Sci. 58, 249–265. 10.1093/gerona/58.3.M249 12634292

[B21] Bonanno G. A. (2004). Loss, trauma, and human resilience: have we underestimated the human capacity to thrive after extremely aversive events? Am. Psychol. 59, 20–28. 10.1037/0003-066X.59.1.20 14736317

[B22] Börsch-Supan A. Brugiavini A. Jürges H. Mackenbach J. Siegrist J. Weber G. (Eds) (2005). Health, Ageing and Retirement in Europe – First Results from the Survey of Health, Ageing and Retirement in Europe. Mannheim: Mannheim Research Institute for the Economics of Aging (MEA).

[B23] Bowling A. (2001). Measuring Disease: A Review of Disease Specific Quality of Life Measurement Scales, 2nd Edn. Buckingham: Open University Press.

[B24] Bowling A. Banister D. Sutton S. Evans O. Windsor J. (2002). A multidimensional model of the quality of life in older age. Aging Ment. Health 6, 355–371. 10.1080/1360786021000006983 12425770

[B25] Bramley T. J. Lerner D. Sarnes M. (2002). Productivity losses related to the common cold. J. Occup. Environ. Med. 44, 822–829. 10.1097/00043764-200209000-00004 12227674

[B26] Braudy Harris P. (2008). Another wrinkle in the debate about successful aging: the undervalued concept of resilience and the lived experience of dementia. Int. J. Aging Hum. Dev. 67, 43–61. 10.2190/AG.67.1.c 18630190

[B27] Brown T. (2006). Confirmatory Factor Analysis for Applied Research. New York, NY: Guilford Press.

[B28] Browne M. W. Cudeck R. (1993). Alternative ways of assessing model fit, in Testing Structural Equations Models, eds Bollen K. A. Long J. S. (Newbury Park, CA: Sage), 136–162.

[B29] Brustia P. Gerino E. Rollè L. (2013). Costruzione dell'identità e sviluppo emotivo nelle coppie gemellari: effetti sul benessere nel ciclo di vita. Psichiatr. Psicoter. 32, 181–196.

[B30] Byrne B. M. (2001). Structural Equation Modeling with AMOS. Rahwah, NJ: Lawrence Erlbaum Associates.

[B31] Byrne G. J. Pachana N. A. (2011). Development and validation of a short form of the geriatric anxiety inventory – the GAI-SF. Int. Psychogeriatr. 23, 125–131. 10.1017/S1041610210001237 20561386

[B32] Cacioppo J. T. Hawkley L. C. Thisted R. A. (2010). Perceived social isolation makes me sad: 5-year cross-lagged analyses of loneliness and depressive symptomatology in the chicago health, aging, and social relations study. Psychol. Aging 25, 453–463. 10.1037/a0017216 20545429 PMC2922929

[B33] Cacioppo J. T. Hughes M. E. Waite L. J. Hawkley L. C. Thisted R. A. (2006). Loneliness as a specific risk factor for depressive symptoms: cross-sectional and longitudinal analyses. Psychol. Aging 21, 140–151. 10.1037/0882-7974.21.1.140 16594799

[B34] Carstensen L. L. Fung H. H. Charles S. T. (2003). Socioemotional selectivity theory and the regulation of emotion in the second half of life. Motiv. Emot. 27, 103–123. 10.1023/A:1024569803230

[B35] Cattan C. White M. Bond J. Learmouth A. (2005). Preventing social isolation and loneliness among older people: a systematic review of health promotion interventions. Ageing Soc. 25, 41–67. 10.1017/S0144686X04002594 27736564

[B36] Charney D. S. (2004). Psychobiological mechanisms of resilience and vulnerability: implications for successful adaptation to extreme stress. Am. J. Psychiatry 161, 195–216. 10.1176/appi.ajp.161.2.195 14754765

[B37] Cicirelli V. G. (2013). Sibling Relationships Across the Life Span. New York, NY: Springer Science & Business Media.

[B38] Connor K. M. Zhang W. (2006). Resilience: determinants, measurement, and treatment responsiveness. CNS Spectr 11, (10 Suppl. 12), 5–12. 10.1017/S1092852900025797 17008825

[B39] De Girolamo G. Rucci P. Scocco P. Becchi A. Coppa F. D'Addario A.. (2000). Quality of life assessment: validation of the Italian version of the WHOQOL-Brief. Epidemiol. Psichiatr. Soc. 9, 45–55. 10.1017/S1121189X00007740 10859875

[B40] de Jager C. A. Budge M. M. Clarke R. (2003). Utility of TICS-M for the assessment of cognitive function in older adults. Int. J. Geriatr. Psychiatry 18, 318–324. 10.1002/gps.830 12673608

[B41] DeSalvo K. B. Bloser N. Reynolds K. He J. Muntner P. (2006). Mortality prediction with a single general self-rated health question. A meta-analysis. J. Gen. Intern. Med. 21, 267–275. 10.1111/j.1525-1497.2005.00291.x 16336622 PMC1828094

[B42] Dickens A. P. Richards S. H. Greaves C. J. Campbell J. L. (2011). Interventions targeting social isolation in older people: a systematic review. BMC Public Health 11:647. 10.1186/1471-2458-11-647 21843337 PMC3170621

[B43] Djukanovic I. Sorjonen K. Petersona U. (2015). Association between depressive symptoms and age, sex, loneliness and treatment among older people in Sweden. Aging Ment. Health 19, 560- 568. 10.1080/13607863.2014.962001 25266255

[B44] Ekwall A. K. Sivberg B. Hallberg I. R. (2005). Loneliness as a predictor of quality of life among older caregivers. J. Adv. Nurs. 49, 23–32. 10.1111/j.1365-2648.2004.03260.x 15610378

[B45] Eurostat (2015a). People in the EU – Statistics on Demographic Changes. Avaliable online at: http://ec.europa.eu/eurostat/statistics-explained/index.php/People_in_the_EU_%E2%80%93_statistics_on_demographic_changes

[B46] Eurostat (2015b). People in the EU – Statistics on an Ageing Society. Avaliable online at: http://ec.europa.eu/eurostat/statistics-explained/index.php/People_in_the_EU_%E2%80%93_statistics_on_an_ageing_society

[B47] Fernández-Ballesteros R. (2003). Light and dark in the psychology of human strengths: The example of psychogerontology, in A Psychology of Human Strengths: Fundamental Questions and Future Directions for a Positive Psychology eds Aspinwal K. G. Staudinger U. M. (Washington, DC: American Psychological Association), 131–147.

[B48] Fernández-Ballesteros R. (2008). Active Aging. The contribution of Psychology. Göttingen: Hogrefe and Huber.

[B49] Findlay R. (2003). Interventions to reduce social isolation amongst older people: where is the evidence? Ageing Soc. 23, 647–658. 10.1017/S0144686X03001296

[B50] Fokkema C. M. Van Tilburg T. G. (2007). Zin en onzin van eenzaamheidsinterventies bij ouderen [Loneliness interventions among older adults: sense or nonsense?]. Tijdschr. Gerontol. Geriatr. 38, 185–203. 10.1007/BF03074846 17879823

[B51] Forsman A. K. Nordmyr J. Wahlbeck K. (2011). Psychosocial interventions for the promotion of mental health and the prevention of depression among older adults. Health Promot. Int. 26, i85–i107. 10.1093/heapro/dar074 22079938

[B52] Fredrickson B. L. Tugade M. M. Waugh C. E. Larkin G. R. (2003). What good are positive emotions in crisis? A prospective study of resilience and emotions following the terrorist attacks on the United States on September 11th, 2001. J. Pers. Soc. Psychol. 84, 365–376. 10.1037/0022-3514.84.2.365 12585810 PMC2755263

[B53] Fry P. S. (2001). Predictors of health quality life perspectives, self-esteem, and life satisfactions of older adults following spousal loss: an 18-month follow-up study of widows and widowers. Gerontologist 41, 787–798. 10.1093/geront/41.6.787 11723347

[B54] Fry P. S. Debats D. L. (2002). Self-efficacy beliefs as predictors of loneliness and psychological distress in older adults. Int. J. Aging Hum. Dev. 55, 233–269. 10.2190/KBVP-L2TE-2ERY-BH26 12693547

[B55] Fry P. S. Debats D. L. (2006). Sources of life strengths as predictors of late-life mortality and survivorship. Int. J. Aging Hum. Dev. 62, 303–334. 10.2190/3VAT-D77G-VCNQ-6T61 16739467

[B56] Fry P. S. Debats D. L. (2010). Sources of human life-strengths, resilience, and health, in eds New Frontiers in Resilient Aging: Life-Strengths and Well-Being in Late Life, eds Fry P. S. Keyes C. L. M. (Cambridge: Cambridge University Press), 15–59.

[B57] Fry P. S. Keyes C. L. M. (2010). Introduction, in New Frontiers in Resilient Aging: Life-Strengths and Well-Being in Late Life, eds Fry P. S. Keyes C. L. M. (Cambridge: Cambridge University Press), 1–14.

[B58] Gabriel Z. Bowling A. (2004). Quality of life from the perspectives of older people. Ageing Soc. 24, 675–691. 10.1017/S0144686X03001582

[B59] Gattuso S. (2003). Becoming a wise old woman: resilience and wellness in later life. Health Sociol. Rev. 12, 171–177. 10.5172/hesr.12.2.171

[B60] Gerino E. Marino E. Brustia P. Lyrakos D. G. Rollè L. (2015). Quality of life in the third age: a research on risk and protective factors. Proc. Soc. Behav. Sci. 187, 217–222. 10.1016/j.sbspro.2015.03.041

[B61] Golden J. Conroy R. M. Bruce I. Denihan A. Green A. Kirby M.. (2009). Loneliness, social support networks, mood and wellbeing in community-dwelling elderly. Int. J. Geriatr. Psychiatry 24, 694–700. 10.1002/gps.2181 19274642

[B62] Ha T. H. (2016). Development of the structural model of middle-aged men's subjective quality of life. J. Digital Convergence 14, 125–135. 10.14400/JDC.2016.14.5.125

[B63] Hagan R. Manktelow R. Taylor B. Mallet J. (2014). Reducing loneliness amongst older people: a systematic search and narrative review. Aging Mental Health 18, 683–693. 10.1080/13607863.2013.875122 24437736

[B64] Hartling L. M. (2008). Strengthening resilience in a risky world: it's all about relationships. Women Ther. 31, 51–70. 10.1080/02703140802145870

[B65] Hawkley L. C. Cacioppo J. T. (2010). Loneliness matters: a theoretical and empirical review of consequences and mechanisms. Ann. Behav. Med. 40, 218–227. 10.1007/s12160-010-9210-8 20652462 PMC3874845

[B66] Hawkley L. C. Thisted R. A. Cacioppo J. T. (2009). Loneliness predicts reduced physical activity: cross-sectional & longitudinal analyses. Health Psychol. 28, 354–363. 10.1037/a0014400 19450042 PMC2791498

[B67] Hildon Z. Montgomery S. M. Blane D. Wiggins R. D. Netuveli G. (2010). Examining resilience of quality of life in the face of health-related and psychosocial adversity at older ages: what is “right” about the way we age? Gerontologist 50, 36–47. 10.1093/geront/gnp067 19549715

[B68] Hoyl M. T. Alessi C. A. Harker J. O. Josephson K. R. Pietruszka F. M. Koelfgen M.. (1999). Development and testing of a five-item version of the geriatric depression scale. J. Am. Geriatr. Soc. 47, 873–878. 10.1111/j.1532-5415.1999.tb03848.x 10404935

[B69] Hu L. Bentler P. M. (1999). Cutoff criteria for fit indexes in covariance structure analysis: conventional criteria versus new alternatives. Struct. Equ. Model. 6, 1–55. 10.1080/10705519909540118

[B70] Inui T. S. (2003). The need for an integrated biopsychosocial approach to research on successful aging. Ann. Intern. Med. 139, 391–394. 10.7326/0003-4819-139-5_Part_2-200309021-00002 12965963

[B71] Jerusalem M. Schwarzer R. (1986). Selbstwirksamkeit [Self-efficacy], in Skalen zur Befindlichkeit und Persönlichkeit. Research Report No. 5, ed Schwarzer R. (Berlin: Freie Universität, Institut für Psychologie), 15–28.

[B72] Jerusalem M. Schwarzer R. (1992). Self-efficacy as a resource factor in stress appraisal processes, in Self-Efficacy: Thought Control of Action, ed Schwarzer R. (Washington, DC: Hemisphere), 195–213.

[B73] Jeste D. V. Savla G. N. Thompson W. K. Vahia I. V. Glorioso D. K. Martin A. S. (2013). Older age is associated with more successful aging: role of resilience and depression. Am. J. Psychiatry 170, 188–196. 10.1176/appi.ajp.2012.12030386 23223917 PMC3593664

[B74] Kline R. B. (2005). Principles and Practice of Structural Equation Modeling, 2nd Edn, New York, NY: The Guilford Press.

[B75] Krause N. (1994). Stressors in salient social roles and well-being in later life. J. Gerontol. 49, 137–148. 10.1093/geronj/49.3.P137 8169343

[B76] Laditka S. B. Corwin S. J. Laditka J. Liu R. Tseng W. Wu B.. (2009). Attitudes about aging well among a diverse group of older Americans: implications for promoting cognitive health. Gerontologist 49, S30–S39. 10.1093/geront/gnp084 19525215

[B77] Lamond A. J. Depp C. A. Allison M. Langer R. Reichstadt J. Moore D. J.. (2009). Measurement and predictors of resilience among community-dwelling older women. J. Psychiatr. Res. 43, 148–154. 10.1016/j.jpsychires.2008.03.007 18455190 PMC2613196

[B78] Lawton M. P. (1991). A multidimensional view of quality of life in frail elders, in The Concept and Measurement of Quality of Life in the Frail Elderly, eds Birren J. E. Lubben J. E. Rowe J. C. Deutchman D. E. (San Diego, CA: Academic Press), 3–27.

[B79] Lawton M. P. Moss M. Hoffman C. Grant R. Ten Have T. Kleban M. H. (1999). Health, valuation of life, and the wish to live. Gerontologist 39, 406–416. 10.1093/geront/39.4.406 10495578

[B80] LeRoy A. S. Murdock K. W. Jaremka L. M. Loya A. Fagundes C. P. (2017). Loneliness Predicts Self-Reported Cold Symptoms After a Viral Challenge. Health psychology: official journal of the Division of Health Psychology; American Psychological Association. 28358524 10.1037/hea0000467 PMC5486976

[B81] Liu L.-J. Guo Q. (2007). Loneliness and health-related quality of life for the empty nest elderly in the rural area of a mountainous county in China. Quality Life Res. 16, 1275–1280. 10.1007/s11136-007-9250-0 17703375

[B82] Lloyd M. Ramon S. Vakalopoulou A. Videmšek P. Meffan C. Roszczynska-Michta J. (2017). Women's experiences of domestic violence and mental health: findings from a European empowerment project. Psychol. Viol. 7, 478–487. 10.1037/vio0000111

[B83] Lucas-Carrasco R. (2012). The WHO quality of life (WHOQOL) questionnaire: Spanish development and validation studies. Quality Life Res. 21, 161–165. 10.1007/s11136-011-9926-3 21611868

[B84] Luszczynska A. Gutiérrez-Do-a B. Schwarzer R. (2005). General self-efficacy in various domains of human functioning: evidence from five countries. Int. J. Psychol. 40, 80–89. 10.1080/00207590444000041

[B85] MacLeod S. Musich S. Hawkins K. Alsgaard K. Wicker E. R. (2016). The impact of resilience among older adults. Geriatr. Nurs. 37, 266–272. 10.1016/j.gerinurse.2016.02.014 27055911

[B86] Marshall G. N. (1991). A multidimensional analysis of internal health locus of control beliefs: separating the wheat from the chaff? J. Pers. Soc. Psychol. 61, 483–491. 10.1037/0022-3514.61.3.483 1941520

[B87] Masi C. M. Chen H. Y. Hawkley L. C. Cacioppo J. T. (2011). A meta-analysis of interventions to reduce loneliness. Person. Soc. Psychol. Rev. 15, 219–266. 10.1177/1088868310377394 20716644 PMC3865701

[B88] Montross L. P. Depp C. Daly J. Reichstadt J. Golshan S. Moore D.. (2006). Correlates of self-rated successful aging among community-dwelling older adults. Am. J. Geriatr. Psychiatry 14, 43–51. 10.1097/01.JGP.0000192489.43179.31 16407581

[B89] National Board of Health Welfare, Sweden (2008). Older People's Mental Health. [In Swedish]. Stockholm Avaliable online at: http://www.socialstyrelsen.se/publikationer2008/2008-131-20

[B90] Nilsson G. Ohrvik J. Lonnberg I. Hedberg P. (2011). Low Psychological General Well-Being (PGWB) is associated with deteriorated 10-year survival in men but not in women among the elderly. Arch. Gerontol. Geriatr. 52, 167–171. 10.1016/j.archger.2010.03.010 20381888

[B91] O'Dwyer S. Moyle W. van Wyk S. (2013). Suicidal ideation and resilience in family careres of people with dementia: a pilot qualitative study. Aging Mental Health 17, 753–760. 10.1080/13607863.2013.789001 23611756

[B92] O'Rourke N. Kupferschmidt A. L. Claxton A. Smith J. Z. Chappell N. Beattie B. L. (2010). Psychological resilience predicts depressive symptoms among souses of person with Alzheimer's disease over time. Aging Mental Health 14, 984–993. 10.1080/13607863.2010.501063 21069604

[B93] Peplau L. A. Perlman D. (1982). Perspectives on loneliness, in Loneliness: A Sourcebook of Current Theory, Research and Therapy, eds Peplau A. L. Perlman D. (New York, NY: John Wiley & Sons), 1–18.

[B94] Peveri L. (2010). Resilienza e Regolazione delle Emozioni. Un Approccio Multimodale. Available onlin at: http://boa.unimib.it/bitstream/10281/7893/3/phd_unimib_707899.pdf

[B95] Prieto-Flores M. E. Forjaz M. J. Fernandez-Mayoralas G. Rojo-Perez F. Martinez-Martin P. (2011). Factors associated with loneliness of noninstitutionalized and institutionalized older adults. J. Aging Health 23, 177–194. 10.1177/0898264310382658 20881107

[B96] Prino L. E. Rollè L. Sechi C. Patteri L. Ambrosoli A. Caldarera A. M.. (2016). Parental relationship with twins from pregnancy to 3 months: the relation among parenting stress, infant temperament, and well-being. Front. Psychol. 7:1628. 10.3389/fpsyg.2016.01628 27818641 PMC5073235

[B97] Reichstadt J. Depp C. A. Palinkas L. A. Folsom D. P. Jeste D. V. (2007). Building blocks of successful aging: a focus group study of older adults' perceived contributors to successful aging. Am. J. Geriatr. Psychiatry 15, 194–201. 10.1097/JGP.0b013e318030255f 17322132

[B98] Rinaldi P. Mecocci P. Benedetti C. Ercolani S. Bregnocchi M. Menculini G.. (2003). Validation of the five-item geriatric depression scale in elderly subjects in three different settings. J. Am. Geriatr. Soc. 51, 694–698. 10.1034/j.1600-0579.2003.00216.x 12752847

[B99] Russell D. W. (1996). UCLA loneliness scale (Version 3): reliability, validity, and factor structure. J. Pers. Assess. 66, 20–40. 10.1207/s15327752jpa6601_2 8576833

[B100] Ryff C. D. Singer B. (2000). Interpersonal Flourishing: a positive health agenda for the New Millennium. Pers. Soc. Psychol. Rev. 4, 30–44. 10.1207/S15327957PSPR0401_4

[B101] Sachs-Ericsson N. Kendall-Tacket K. A. Sheffler J. Arce D. Rushing N. C. Cosentino E. (2014). The influence of prior rape on the psychological and physical health functioning of older adults. Aging Mental Health 18, 717–730. 10.1080/13607863.2014.884538 24521090

[B102] Schoevers R. A. Beekman A. T. Van Tillburg W. Deeg D. J. Jonker C. Geerlings M. I.. (2000). Association of depression and gender with mortality in old age. Results from the Amsterdam Study of the Elderly (AMSTEL). Br. J. Psychiatry 177, 336–342. 10.1192/bjp.177.4.336 11116775

[B103] Schumacker R. E. Lomax R. G. (1996). A Beginner s Guide to Structural Equation Modeling. Mahwah, NJ: Erlbaum.

[B104] Settersten R. A. Godlewski B. (2016). Concepts and theories of age and aging, in Handbook of Theories of Aging, 3rd Edn, eds Bengtson V. L. Settersten R. A. (New York, NY: Spriger Publishing Company, LLC), 9–26.

[B105] Sharpley C. F. Yardley P. (1999). The relationship between cognitive hardiness, explanatory style, and depression-happiness in post-retirement men and women. Aust. Psychol. 34, 198–203. 10.1080/00050069908257454

[B106] Sibilia L. Schwarzer R. Jerusalem M. (1995). Italian Adaptation of the General Self-Efficacy Scale. Available online at: http://userpage.fu-berlin.de/~health/italian.htm

[B107] Smith G. C. Kohn S. J. Savage-Stevens S. E. Finch J. J. Ingate R. Lim Y. (2000). The effects of interpersonal and personal agency on perceivedcontrol and psychological well-being in adulthood. Gerontologist 40, 458–468. 10.1093/geront/40.4.458 10961035

[B108] Smith J. L. Hollinger-Smith L. (2015). Savoring, resilience, and psychological well-being in older adults. Aging Ment. Health 19, 192–200. 10.1080/13607863.2014.986647 25471325

[B109] Smith-Osborne A. Felderhoff B. (2016). Formal and family caregiver protective factors in systems of care: a systematic review with implications toward a resilience model for aging veterans. Traumatology 22, 29–39. 10.1037/trm0000056

[B110] Stephens C. Breheny M. Mansvelt J. (2015). Healthy ageing from the perspective of older people: a capability approach to resilience. Psychol. Health 30, 715–731. 10.1080/08870446.2014.904862 24678916

[B111] Theeke A. L. (2010). Sociodemographic and health-related risks for loneliness and outcome differences by loneliness status in a sample of U.S. older adults. Res. Gerontol. Nurs. 3, 113–125. 10.3928/19404921-20091103-99 20415360

[B112] Theeke A. L. (2012). Older people who report loneliness have increased risk of mortality and functional decline. Evid Based Nurs. 172, 1078–1083. 10.1136/eb-2012-101052 23190457

[B113] Tiikkainen P. Heikkinen R. L. (2005). Associations between loneliness, depressive symptoms and perceived togetherness in older people. Aging Mental Health 9, 526–534. 10.1080/13607860500193138 16214700

[B114] United Nations (UN) (2002). World Population Prospects: The 2000 Revision. Analytical report. United Nations, New York, NY.

[B115] Vahia I. V. Thompson W. K. Depp C. A. Allison M. Jeste D. V. (2012). Developing a dimensional model for successful cognitive and emotional aging. Int. Psychogeriatr. 24, 515–523. 10.1017/S1041610211002055 22050770

[B116] Wagnild G. (2009). A review of the resilience scale. J. Nurs. Meas. 17, 105–113. 10.1891/1061-3749.17.2.105 19711709

[B117] Wagnild G. M. Young H. G. (1993). Development and psychometric evaluation of the resilience scale. J. Nurs. Meas. 1, 165–178. 7850498

[B118] Wallace K. A. (2003). An examination of hardiness in male older adults living in a rural setting, in Advances in Psychology Research, Vol. 23, ed Shovov S. P. (Hauppauge: Nova Science Publishers), 78–94.

[B119] Wild K. Wiles J. L. Allen R. E. S. (2013). Resilience: thoughts on the value of the concept for critical gerontology. Aging Soc. 33, 137–158. 10.1017/S0144686X11001073

[B120] Wiles J. L. Wild K. Kerse N. Allen R. E. S. (2012). Resilience from the point of view of older people: “there's still life beyond a funny knee. Soc. Sci. Med. 74, 416–424. 10.1016/j.socscimed.2011.11.005 22204841

[B121] Wong N. Liu H. Lin C. Huang C. Wai Y. Lee S.. (2016). Loneliness in late-life depression: structural and functional connectivity during affective processing. Psychol. Med. 46, 2485–2499. 10.1017/S0033291716001033 27328861

[B122] World Health Organization (WHO) (1993). Measuring Quality of Life: The Development of the World Health Organization Quality of Life Instrument (WHOQOL). WHO, Geneva.

[B123] World Health Organization (WHO) (2002). Active Ageing: a Policy Framework. World Health Organization, Geneva Avaliable online at: http://whqlibdoc.who.int/hq/2002/who_nmh_nph_02.8.pdf (Accessed April 19, 2017).

[B124] World Health Organization (WHO) (2015). World Report on Aging and Health. World Health Organization, Geneva. Avaliable onlne at: http://apps.who.int/iris/bitstream/10665/186463/1/9789240694811_eng.pdf (Accessed April 19, 2017).

